# ADSC Exosomes Mediate lncRNA-MIAT Alleviation of Endometrial Fibrosis by Regulating miR-150-5p

**DOI:** 10.3389/fgene.2021.679643

**Published:** 2021-06-09

**Authors:** Xiaowen Shao, Jinlong Qin, Chendong Wan, Jiajing Cheng, Lian Wang, Guihai Ai, Zhongping Cheng, Xiaowen Tong

**Affiliations:** ^1^Department of Obstetrics and Gynecology, Shanghai Tenth People’s Hospital, Tongji University School of Medicine, Shanghai, China; ^2^Department of Obstetrics and Gynecology, Fourth People’s Hospital of Yixing City, Wuxi, China; ^3^Department of Obstetrics and Gynecology, Tongji Hospital, Tongji University School of Medicine, Shanghai, China

**Keywords:** endometrial fibrosis, ADSC, ADSC-exosomes, LncRNA-MIAT, miR-150-5p

## Abstract

**Background:**

Secondary infertility remains a major complication of endometrial fibrosis in women. The use of exosomes from adipose-derived mesenchymal stem cells (ADSCs) has shown promising results for the treatment of endometrial fibrosis. However, the mechanisms of action of ADSC-exosome (ADSC-Exo) therapy remain unclear.

**Materials and Methods:**

An endometrial fibrosis model was established in mice treated with alcohol and endometrial epithelial cells (ESCs) treated with TGF-β1. ADSCs were isolated from Sprague Dawley (SD) rats, and exosomes were isolated from ADSCs using ExoQuick reagent. Exosomes were identified by transmission electron microscopy (TEM), NanoSight, and Western blot analysis. The expression level of lncRNA-MIAT was detected by qPCR analysis. Western blot analysis was carried out to determine the protein levels of fibrosis markers (TGFβR1, α-SMA, and CK19). A dual-luciferase reporter gene assay was used to verify the relationship between target genes. The endometrial tissues of the endometrial fibrosis model were stained with HE and Masson’s trichrome.

**Results:**

ADSCs and ADSC-Exos were successfully isolated, and the expression level of lncRNA-MIAT was significantly down-regulated in endometrial tissue and the TGF-β1-induced ESC injury model, whereas ADSC-Exos increased the expression of lncRNA-MIAT in the TGF-β1-induced ESC model. Functionally, ADSC-Exo treatment repressed endometrial fibrosis *in vivo* and *in vitro* by decreasing the expression of hepatic fibrosis markers (α-SMA and TGFβR1) and increasing the expression of CK19. Moreover, miR-150-5p expression was repressed by lncRNA-MIAT in the TGF-β1-induced ESC injury model. The miR-150-5p mimic promoted TGF-β1-induced ESC fibrosis.

**Conclusion:**

ADSC-Exos mediate lncRNA-MIAT alleviation of endometrial fibrosis by regulating miR-150-5p, which suggests that lncRNA-MIAT from ADSC-Exos may be a viable treatment for endometrial fibrosis.

## Introduction

Endometrial fibrosis is a catastrophic condition that causes secondary infertility, which is related to lower implantation and pregnancy rates. Excessive uterine curettage, endometrial tuberculosis, and other factors can lead to endometrial fibrosis. Various strategies have been suggested to treat endometrial fibrosis, such as immunotherapy and endometrial stimulation by biopsy, but none have been validated to date (Hooker et al., 2016). However, the treatment strategy for endometrial fibrosis remains to be investigated.

Clinical studies have confirmed that stem cell therapy is a promising therapeutic approach for tissue damage (Ding et al., 2014; Duncan and Valenzuela, 2017; Azizi et al., 2018; Cao et al., 2018; Zhu et al., 2019; Han H. S. et al., 2020). Stem cell therapy has many limits due to low cell survival. In recent years, a number of studies have confirmed that stem cell exocrine signaling plays a significant role in tissue repair, including skin wound healing (Pan and Johnstone, 1983), myocardial injury repair (Johnstone, 2006), nerve injury repair (Phinney and Pittenger, 2017), bone and cartilage regeneration (Pu et al., 2017), and repair after liver (Waters et al., 2018) and kidney injury (Farinazzo et al., 2015). Some studies have reported that ADSC exosomes regulate the process of endometrial fibrosis. The molecular mechanism of ADSC-Exos is not well studied and is the focus of intensive research on secondary infertility (Koh et al., 2016; Eirin et al., 2017; Ashmwe et al., 2018).

Recently, the biological function of exocrine long noncoding RNAs (lncRNAs) has attracted much attention (Gupta et al., 2013; Di Pietro, 2016; Lugea and Waldron, 2017; Whiteside, 2017; Wan et al., 2018). LncRNAs have been shown to play critical roles in the regulation of biological processes, including cellular apoptosis, gene regulation, and cancer development (Kooijmans et al., 2012; Ferguson and Nguyen, 2016; Barile and Vassalli, 2017; Eirin et al., 2017; Shahabipour et al., 2017; Han et al., 2019). LncRNAs have also been shown to improve various diseases. However, there is little research on the role of lncRNAs from ADSC-Exos in endometrial fibrosis. miRNAs are noncoding single-stranded small RNAs with a length of approximately 22 nucleotides (Rarani et al., 2018; Guo et al., 2021). miRNAs participate in essential functions in various biological regulatory pathways including cell apoptosis, differentiation, and proliferation (Kanekura et al., 2016; Klinge, 2018). In recent years, more and more studies have shown that lncRNAs can exert their biological functions by regulating the expression of miRNAs, thus affecting the occurrence and development of uterine diseases (Ghafouri-Fard et al., 2020; Thankachan et al., 2021). Several studies have demonstrated that lncRNAs may be involved in the formation of competitive endogenous RNA (ceRNA) regulatory networks, and there may be a negative correlation between lncRNA and miRNA (Vallone et al., 2018).

In the present study, we found that lncRNA-MIAT from ADSC-Exos plays a crucial role in endometrial fibrosis progression. Additionally, the therapeutic effects of artificial lncRNA-MIAT were confirmed in both *in vitro* and *in vivo* models. It was speculated that lncRNA-MIAT possibly accelerated the progression of endometrial cancer by impeding miR-150-5p expression. Thus, activation of lncRNA-MIAT could emerge as a novel therapy for endometrial fibrosis.

## Materials and Methods

### Adipose-Derived Stem Cell Isolation and Culture

A SD rat was sterilized after routine anesthesia. The groin adipose tissue was cut into a paste, the blood was removed by washing with PBS, 0.075% collagenase digestion was used to remove the matrix, normal saline was used to stop collagenase digestion, and, after centrifugation, the supernatant and undigested fat were removed. The cells were resuspended in DMEM supplemented with 10% FBS, centrifuged, washed, and counted under a microscope. A total of 10^4^ cells/ml were plated in a culture dish, and cultured in a 5% CO_2_ incubator at a constant temperature. The first change of medium took place 4 h later. After 3 days, the cells were digested and passaged after reaching 80% confluency.

### ADSC-Exosomes Preparation

Ten milliliters of culture supernatant was collected by a high-speed cryopreservation centrifuge at 4°C for 15 min. Impurities were removed from the supernatant, the supernatant was transferred to another sterilized 15 mL centrifuge tube, and 2 mL of ExoQuick reagent (EXOQ10TC) was added to the tube. The tube was mixed upside down and then incubated overnight in a refrigerator at 4°C. Then, the mixture was centrifuged at 4°C at 30000 *g* for 30 min. White or beige precipitation at the bottom of the centrifuge tube was visible, and the supernatant was transferred to another tube for later experiments. Then, 100 μL PBS or sterile water was added after heavy suspension precipitation and the samples were stored in the refrigerator at −80°C.

### ADSC-Exosomes Characterization

A kit from SBI was used to extract the exocrine from the supernatant, and four samples were resuspended in 100 μL PBS. One part of each sample was mixed with 2.5% glutaraldehyde to fix the exocrine and was then homogenized. The samples were dehydrated with 1% nitrous oxide treatment, and the exosome size and morphology were observed by transmission electron microscopy (TEM). Ten microliters of the other part of each sample was adsorbed onto a mica sheet, and the mica sheet was dried and fixed in a desiccator and then washed with PBS. After nitrogen drying, the exocrine on the mica sheet was scanned using a 5500 series (atomic force microscopy, AFM) atomic force microscope mode. One microlitre of exocrine suspension was diluted 1000 times in 1 mL PBS, the liquid was injected into the instrument tank with a needle, and Nanosight LM10 nanotracking technology was used to measure the exosome particle size and concentration of exocrine. Western blot analysis of protein in the exosomes (CD63) was conducted, and the following primary antibody was used: CD63 (1:1000, Proteintech).

### Culture of Endometrial Epithelial Cells (ESCs) and TGF-β1 Treatment

The inner membrane tissue was shredded, digested with trypsin-EDTA, and filtered through a mesh screen. The epithelial and interstitial cells were separated by a 400 mesh (38 μm pore size) screen filter. The filtrate was collected and centrifuged (10 cm, 1000 r/min, for 10 min), the supernatant was centrifuged, and the backwash solution mainly contained the inner membrane epithelial cell mass. The precipitated epithelial cell mass was collected and inoculated with culture medium (plus two antibodies). After heavy resuspension, the cells were counted and placed in a 37°C incubator at 5% CO_2_ with humidity. After 24 h, the culture medium was changed.

ESCs were treated with 10 μg/L TGF-β1 (Sigma-Aldrich, United States) in DMEM supplemented with 1% FBS for 48 h.

### Quantitative Real Time-PCR (qPCR)

Total RNA was extracted from cell lines using TRIzol reagent (Invitrogen). Five hundred nanograms of RNA from each sample was reverse transcribed using a SuperScript RT kit (Invitrogen, Carlsbad, CA, United States). qPCR was performed on an Applied Biosystems 7300 Real-time PCR system (Applied Biosystems, CA) using SYBR Premix Ex TaqTM (Takara, Japan). β-actin or U6 were used as internal references. The relative expression of target genes was calculated using the 2 ^−ΔΔCt^ method. All primers employed were the following: MIAT (sense): CAGCCTCAAACCCAGGGC; MIAT (antisense): CGCAGGACTGTTGTGCCA; *β-actin* (sense): AGGTCATCACTATTGGCAACGA; *β-actin* (antisense): CCAAGAAGGAAGGCTGGAAAA.

### Establishment of Endometrial Injury Model

The Institutional Ethics Committee of Tongji Hospital, affiliated with the Tongji University, approved the animal experiment protocol. Mature and unmated female mice were selected, aged 8–10 weeks and weighing 22–25 g, and housed at a constant temperature (22°C). Five days before surgery, each mouse was subcutaneously injected with progesterone (3 mg/kg), and vaginal pictures were observed daily to synchronize the oestrus cycle of all mice. An endometrial fibrosis model was established in mice by perfusing 95% ethanol into the right side of the uterus. The mice were divided into five groups. In the sham group, the uterine horn was sutured only after longitudinal incision without alcohol injury to the endometrium. Mice in the model group were not treated after endometrial injury. Mice in the exosomes group received an injection of exosomes (5 μg/mouse) into the damaged uterine cavity. In the miR-150 group, miR-150-5p agomir (5 nmol/mouse) were injected into the damaged area of endometrial tissue.

### Functional Identification of Pregnancy

Endometrial-injury mice were treated as described in the above groups and were paired with male mice in cages (male and female ratio 5:2) to evaluate the relationship between endometrial injury and pregnancy rate.

### Endometrial Morphology in Each Group

All mice were sacrificed by intraperitoneal injection with overdose of chloral hydrate. Uterine tissues were harvested, immersed in 4% paraformaldehyde, embedded in paraffin, cut into 4 μm sections, stained with haematoxylin/eosin (H&E) and Masson and then examined under a microscope (Olympus, Japan).

### Western Blot Analysis

Total protein was extracted for 30 min with 50 mL radioimmunoprecipitation assay buffer containing 0.5 mL protease inhibitors. Total protein concentrations were quantified by the bicinchoninic acid protein assay. Primary antibodies against CK19 (1:1000; A19040, ABclonal, United States), TGFβR1 (1:1000; ab31013, Abcam, United States) and α-SMA (1:1000; ab7817, Abcam, United States) were used for the analysis. Chemiluminescence measurements and semiquantitative values were obtained by a ChemiDocXRS+Imaging system and Image Gauge V3.12. Protein levels were quantified relative to β-actin.

### Dual-Luciferase Reporter Gene Assay

Predicted binding sequences of lncRNA-MIAT to miR-150-5p and wild-type and mutated full sequences were cloned into the pGL3 vector and named pGL3-lncRNA-MIAT-wild-type (WT) and pGL3-lncRNA-MIAT mutant (MUT), respectively. HEK-293T cells were co-transfected with wild-type or mutant sequences and miR-150-5p mimics/NC for 48 h. the luciferase activity was performed using a dual-luciferase reporter assay system (Promega, United States).

### Statistical Analysis

Each experiment was performed independently at least three times. Data are expressed as means ± standard deviation (SD). Student’s *t*-test was used to compare the difference between two groups. All analyses were run using the SPSS 15.0 statistical package (SPSS Inc., Chicago, IL, United States).

## Results

### Isolation and Characterization of ADSC-Exosomes

**FIGURE 1:**
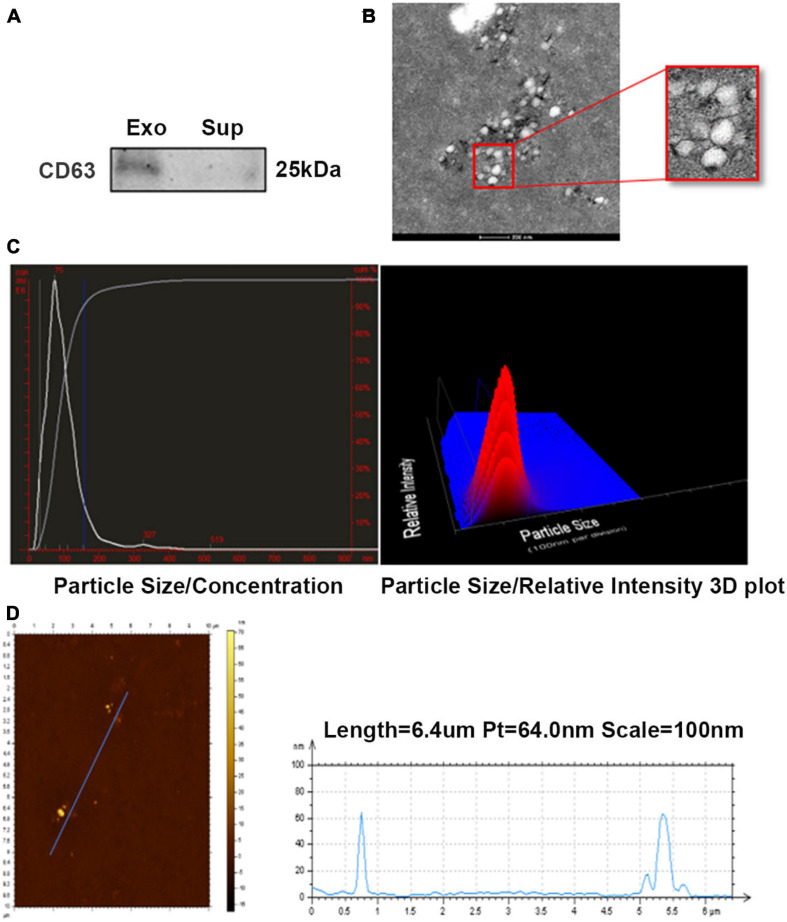
Characterization of ADSC-exosomes. **(A)** Western blot analysis of CD63 expression in ADSC exosomes. **(B)** TEM analysis of exosomes secreted by ADSCs. Scale bar = 200 nm. **(C,D)** The particle size of the exosomes secreted by ADSCs was measured by NanoSight LM10 and AFM5500. ADSC, adipose-derived mesenchymal stem cells; TEM, transmission electron microscopy.

To investigate the effect of ADSC-derived exosomes on endometrial injury, we first identified isolated exosomes. The characterization of ADSC exosomes is shown in Figure 1. Western blot analysis demonstrated that the exosomal marker protein CD63 was present in the ADSC-exosomes (Figure 1A), and TEM analysis showed that exosomes purified from the ADSCs were round, membrane-bound vesicles (Figure 1B). NanoSight LM10 analysis estimated that ADSC-exosomes were between 30 nm and 150 nm in size (Figure 1C). AFM5500 analysis revealed an ADSC-exosome length of 60 nm (Figure 1D). The above results all proved that the substances we extracted could be identified as exosomes.

### LncRNA-MIAT in ADSC-Exos Alleviates Endometrial Fibrosis

**FIGURE 2:**
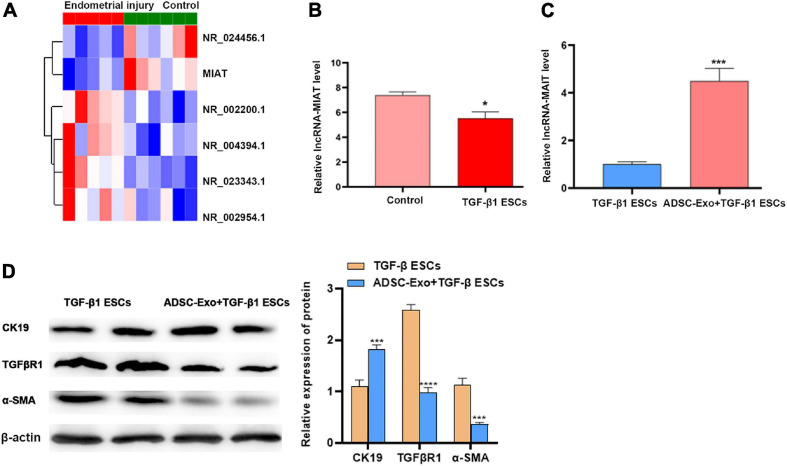
LncRNA-MIAT in ADSC-Exos alleviates endometrial fibrosis. **(A)** Thermal map analysis of the top differentially expressed lncRNAs in endometrial samples from patients with endometrial injury. **(B)** qPCR analysis of the lncRNA-MIAT expression level in TGF-β1-induced ESC injury. **(C)** qPCR analysis of the lncRNA-MIAT expression level in TGF-β1-induced ESC injury cocultured with or without ADSC-Exos. **(D)** Western blot analysis of CK19, TGFβR1, and α-SMA protein expression in TGF-β1-induced ESC injury co-cultured with or without ADSC-Exos. Data are presented as the mean ± SD (*n* = 3). Two-tailed unpaired Student’s *t*-test, *P < 0.05; ***P < 0.001; ****P < 0.0001. ADSC-Exos, adipose-derived mesenchymal stem cells-exosome; qPCR, real time quantitative PCR; TGF-β1, transforming growth factor-β1; ESC, endometrial epithelial cells; CK19, cytokeratin 19; TGFβR1, homo sapiens transforming growth factor beta receptor 1; α-SMA, alpha smooth muscle Actin. SD, standard deviation.

To explore the relationship between healthy controls and patients with endometrial injury, we isolated cells and extracted RNA for sequencing. Simultaneously, the correlation between molecules, inflammatory injury, and fibrosis was further investigated. Through our analysis, we identified 6 differentially expressed long-chain noncoding genes, among which lncRNA-MIAT showed the greatest difference (Figure 2A). Then, the expression of lncRNA-MIAT was quantified using qPCR analysis, and its expression level in TGF-β1-induced-ESC-injury cells was lower than that in ESC controls, which was consistent with our sequencing results (Figure 2B). Previous studies on ADSC-Exos have shown that ADSC-Exos can be used to repair various tissue injuries (Koh et al., 2016; Pu et al., 2017); Thus, we investigated the effect of ADSC-Exos on endometrial injury repair. TGF-β1 was used to construct the ESC injury model *in vitro*. ADSC-Exos were co-cultured with endometrial epithelial injury cells, and the expression of lncRNA-MIAT was quantified by qPCR analysis. As shown in Figure 2C, ADSC-Exos resulted in increased expression of lncRNA-MIAT in TGF-β1-induced ESCs. Fibrosis markers (TGFβR1, α-SMA, and CK19) were detected by Western blot analysis to investigate the effect of ADSC-Exos on the fibrosis of endometrial epithelial cells. As shown in Figure 2D, the protein expression levels of TGFβR1 and α-SMA were significantly down-regulated, and CK19 expression was up-regulated in the co-culture of ADSC-Exos and TGF-β1-induced ESCs. These results confirmed that ADSC-Exos could mediate lncRNA-MIAT to alleviate fibrosis following endometrial injury.

### LncRNA-MIAT in ADSC-Exos Alleviates Endometrial Fibrosis by Targeting miR-150 *in vitro*

**FIGURE 3:**
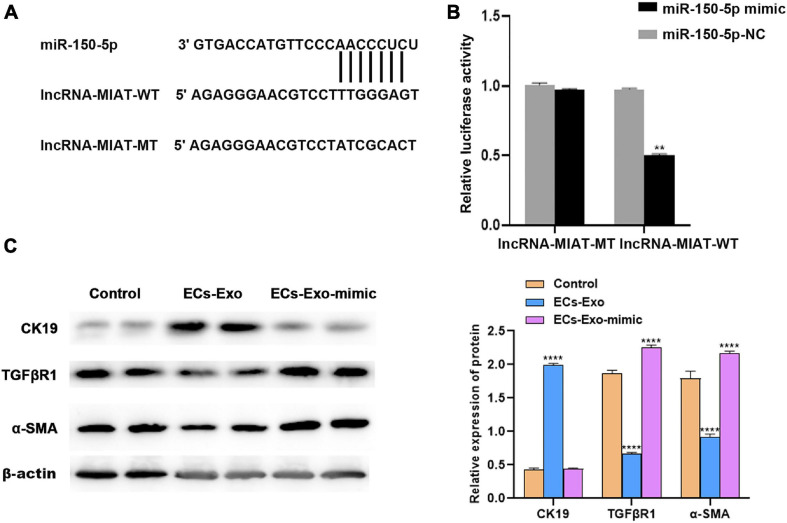
LncRNA-MIAT in ADSC-Exos alleviates endometrial fibrosis by targeting miR-150. **(A)** Schematic representation of the miR-150 site in lncRNA-MIAT-3’UTR. **(B)** Luciferase activity was assayed in TGF-β1-induced ESC injury co-transfected with miR-150 and luciferase reporters containing lncRNA-MIAT-3’UTR. **(C)** Western blot analysis of CK19, TGFβR1, and α-SMA protein expression in TGF-β1-induced ESC injury co-cultured with or without ADSC-Exos in the absence or presence of miR-150 mimic. Data are presented as the mean ± SD (*n* = 3). Two-tailed unpaired Student’s *t*-test, **P < 0.01; ****P < 0.0001. ADSC-Exos, adipose-derived mesenchymal stem cells-exosome; TGF-β1, transforming growth factor-β1; ESC, endometrial epithelial cells; CK19, cytokeratin 19; TGFβR1, Homo sapiens transforming growth factor beta receptor 1; α-SMA, alpha smooth muscle actin. SD, standard deviation.

Many studies have demonstrated that lncRNAs could play a role in endometrial injury repair by adsorbing miRNAs (Tay et al., 2014; Chen et al., 2018). The starBase v3.0 database was used to predict the potential miRNA binding sites in lncRNA-MIAT. To verify the direct binding of miR-150-5p to lncRNA-MIAT, pGL3-lncRNA-MIAT and pGL3-lncRNA-MIAT-Mut were constructed and co-transfected with miR-150-5p into TGF-β1-induced ESCs (Figure 3A). Figure 3B shows that the pGL3-lncRNA-MIAT-WT luciferase activity was markedly decreased following co-transfection with miR-150-5p than with co-transfection with miR-NC, whereas miR-150-5p did not repress the luciferase activity of pGL3-lncRNA-MIAT-Mut. The effects of lncRNA-MIAT and miR-150-5p on regulating endometrial injury and fibrosis *in vitro* were then assessed. Figure 3C shows that miR-150-5p overexpression in the co-culture of ADSC-Exos and TGF-β1-induced ESCs significantly up-regulated the expression levels of TGFβR1 and α-SMA, whereas it down-regulated the expression of CK19. These results suggested that lncRNA-MIAT in ADSC-Exos could alleviate endometrial fibrosis by targeting miR-150.

### LncRNA-MIAT in ADSC-Exos Alleviates Mouse Endometrial Fibrosis by Targeting miR-150

**FIGURE 4:**
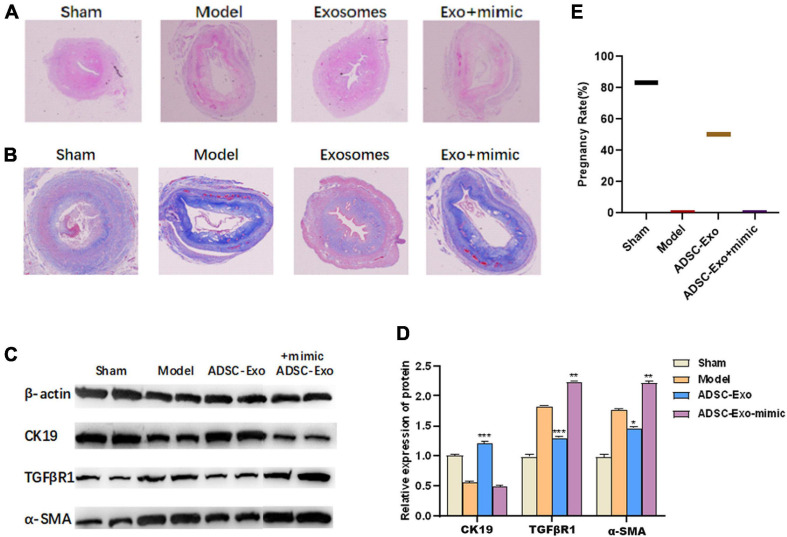
LncRNA-MIAT in ADSC-Exos alleviates mouse endometrial fibrosis by targeting miR-150. **(A)** The number of glands and levels of fibrosis were detected by H&E staining of the endometrial tissue from mouse endometrial fibrosis models (magnification, ×20). **(B)** The number of glands and levels of fibrosis were detected by Masson staining of the endometrial tissue from mouse endometrial fibrosis models (magnification, ×20). **(C)** The pregnancy rate was measured in each group of mouse endometrial fibrosis models. **(D,E)** Western blot analysis of CK19, TGFβR1 and α-SMA protein expression in the endometrial tissue of mouse endometrial fibrosis models. Data are presented as the mean ± SD (*n* = 3). *P < 0.05, **P < 0.01, ***P < 0.001. H&E, hematoxylin-eosin; ADSC-Exos, adipose-derived mesenchymal stem cells-exosome; CK19, cytokeratin 19; TGFβR1, homo sapiens transforming growth factor beta receptor 1; α-SMA, alpha smooth muscle actin. SD, standard deviation.

To evaluate the effects of ADSC-Exos on lncRNA-MIAT associated with endometrial injury, we decided to verify this hypothesis *in vivo*. A mouse endometrial fibrosis model was established using alcohol-induced fibrosis. Compared with the control group, H&E staining showed that the epithelial cells were disordered, the interstitial hyperaemia, endometrial thickness was significantly thinner, and the degree of injury was significantly increased in the model group. Compared with the model group, the endometrial condition after ADSC-Exo treatment was significantly improved in the exosome group, but the endometrial injury was aggravated after miR-150-5p mimic treatment (magnification, ×20, Figure 4A). Meanwhile, Masson’s trichome staining showed that ADSC-Exo inhibited alcohol-induced fibrosis in the uterus, whereas the degree of uterine fibrosis was further aggravated by treatment with the miR-150-5p mimic (magnification, ×20, Figure 4B). In accordance with the *in vitro* results, the overexpression of miR-150-5p significantly up-regulated the expression of TGFβR1 and α-SMA and down-regulated the expression of CK19 in ADSC-Exo-treated endometrial-injury tissues (Figures 4C,D). Moreover, whether ADSC-Exos can improve the pregnancy rate was analyzed. As shown in Figure 4E, mice in the ADSC-Exo group exhibited an increased pregnancy rate (50%), and further treatment with the miR-150-5p mimic reduced the pregnancy rate.

## Discussion

In this study, the effect and mechanism of ADSC- derived exosomes on endometrial fibrosis were further investigated. The current data verified that (1) lncRNA-MIAT in ADSC-Exos alleviates endometrial fibrosis; (2) lncRNA-MIAT in ADSC-Exos alleviates endometrial fibrosis by targeting miR-150 *in vitro*; and (3) lncRNA-MIAT in ADSC-Exos alleviates endometrial fibrosis in mice by targeting miR-150. These results indicate the important role of the ADSC-Exo-lncRNA-MIAT/miR-150-5p axis in regulating endometrial fibrosis and may provide a therapeutic opportunity for patients with endometrial fibrosis.

The pathological manifestation of endometrial fibrosis is the attachment of endometrial glandular epithelium and interstitial fibrous tissue, resulting in a sharp decrease or even complete loss of normal endometrial tissue (Guo, 2020). Therefore, in this study, TGFβR1, α-SMA, and CK19 were used to measure endometrial fibrosis (Li et al., 2016; Salma et al., 2016; Zhu et al., 2017; Yao et al., 2019). Endometrium fibrosis can directly lead to abnormal menstruation, amenorrhea, infertility, seriously affect the patient’s family happiness. So far, there is a lack of good endometrial repair methods in clinic. Therefore, this study aims to explore a new clinical treatment method.

ADSCs have significant effects on the repair of premature ovarian failure (Xie et al., 2017), repair of myocardial injury (Lee et al., 2020), repair of nerve injury (Sadie-Van Gijsen, 2019), regeneration of bone and cartilage (Tiscornia et al., 2006), liver (Liu and Chen, 2018), and kidney (Zuk et al., 2002) after injury. Our previous studies also found that ADSCs transplantation can improve the repair of endometrial injury (Shao et al., 2019). Exosomes are nanoscale vesicles, approximately 30–100 nm in diameter, released into the extracellular environment in the form of exocytosis after fusion of intracellular Multivesicular Bodies (MVBs) with the cell membrane (Xie and Zeng, 2020). Compared with other vectors, exosomes may be a promising therapeutic strategy due to their lower immunogenicity, non-cytotoxicity, and non-mutagenicity to receptors. Therefore, we examined the anti-fibrosis properties of ADSC-EXOS in endometrial injury. The results showed that after ADSC-EXOS treatment of TGF-β1-induced ESCs, the protein expression levels of TGFβR1 and α-SMA were significantly down-regulated, and CK19 expression was up-regulated in the co-culture of ADSC-EXOS and TGF-β1-induced ESCs. However, the molecular mechanism of the anti-fibrosis effect of ADSC-Exos in endometrial fibrosis has not been fully elucidated.

Exosomal metastatic lncRNAs can regulate the proliferation, migration, invasion and apoptosis of tumor cells, and may be used as biomarkers for tumor-related diagnosis and prognosis (Han S. et al., 2020). LncRNA-MIAT was initially discovered as a tumor-associated lncRNA and was reported to regulate splicing and epigenetic control of gene expression. In this study, we found that lncRNA-MIAT was low expressed in endometrial fibrosis tissues and TGF-β1-induced-ESC-injury cells. However, lncRNA-MIAT was highly expressed in ADSC-EXOS, suggesting that lncRNA may play an important role in the process of endometrial fibrosis. Recent studies have shown that lncRNA plays a regulatory role in a variety of fibrosis, including diabetic nephrotic fibrosis, cardiac fibrosis, myocardial fibrosis induced by atrial fibrillation, renal fibrosis, etc. The protein expression levels of TGFβR1 and α-SMA were significantly down-regulated, and CK19 expression was up-regulated in the co-culture of ADSC-Exos and TGF-β1-induced ESCs. These results confirmed that ADSC-Exos could mediate lncRNA-MIAT to alleviate fibrosis following endometrial injury.

In recent years, more and more studies have shown that lncRNAs can exert their biological functions by regulating the expression of miRNAs, thus affecting the occurrence and development of uterine diseases (Ghafouri-Fard et al., 2020; Thankachan et al., 2021). Therefore, the Starbase v3.0 database was used to predict the potential miRNA binding sites in lncRNA-MIAT. The direct binding of miR-150-5p with lncRNA-MIAT was further verified by dual luciferase gene reporter assay. The effects of lncRNA-MIAT and miR-150-5p on regulating endometrial injury and fibrosis *in vitro* and *in vivo* were then assessed. The results suggested that lncRNA-MIAT in ADSC-Exos could alleviate endometrial fibrosis by targeting miR-150.

At present, our research still has some limitations. In the current study, whether lncRNA-MIAT can alleviate fibrosis after endometrial injury remains to be explored. We will conduct further experiments on overexpression or knockout of lncRNA-MIAT.

In summary, our study revealed that lncRNA-MIAT in ADSC-Exos improves endometrial fibrosis by regulating miR-150-5p. This study aimed to offer new insight into the importance of lncRNA-MIAT in ADSC-Exo therapy, and we suggest that the manipulation of lncRNA-MIAT expression may be a promising strategy for the treatment of endometrial fibrosis.

## Data Availability Statement

The raw data supporting the conclusions of this article will be made available by the authors, without undue reservation.

## Ethics Statement

The animal study was reviewed and approved by Animal Ethic Committee of Tongji Hospital Affiliated to Tongji University.

## Author Contributions

All authors listed have made a substantial, direct and intellectual contribution to the work, and approved it for publication.

## Conflict of Interest

The authors declare that the research was conducted in the absence of any commercial or financial relationships that could be construed as a potential conflict of interest.
